# The Prognostic Significance of Sleep and Circadian Rhythm for Myocardial Infarction Outcomes: Case-Control Study

**DOI:** 10.2196/63897

**Published:** 2025-02-04

**Authors:** Wei-Chih Chin, Pao-Hsien Chu, Lung-Sheng Wu, Kuang-Tso Lee, Chen Lin, Chien-Te Ho, Wei-Sheng Yang, I-Hang Chung, Yu-Shu Huang

**Affiliations:** 1 Division of Psychiatry and Sleep Center Chang Gung Memorial Hospital Taoyuan Taiwan; 2 College of Medicine Chang Gung University Taoyuan City Taiwan; 3 College of Life Sciences and Medicine National Tsing Hua University Hsinchu Taiwan; 4 Department of Cardiology Chang Gung Memorial Hospital Taoyuan Taiwan; 5 Department of Biomedical Sciences and Engineering National Central University Taoyuan Taiwan; 6 Department of Psychiatry New Taipei City Tucheng Hospital New Taipei City Taiwan

**Keywords:** myocardial infarction, circadian rhythm, actigraphy, nonparametric analysis, prognosis, sleep, heart rate variability, activity

## Abstract

**Background:**

Myocardial infarction (MI) is a medical emergency resulting from coronary artery occlusion. Patients with acute MI often experience disturbed sleep and circadian rhythm. Most previous studies assessed the premorbid sleep and circadian rhythm of patients with MI and their correlations with cardiovascular disease. However, little is known about post-MI sleep and circadian rhythm and their impacts on prognosis. The use of actigraphy with different algorithms to evaluate sleep and circadian rhythm after acute MI has the potential for predicting outcomes and preventing future disease progression.

**Objective:**

We aimed to evaluate how sleep patterns and disrupted circadian rhythm affect the prognosis of MI, using actigraphy and heart rate variability (HRV). Nonparametric analysis of actigraphy data was performed to examine the circadian rhythm of patients.

**Methods:**

Patients with MI in the intensive care unit (ICU) were enrolled alongside age- and gender-matched healthy controls. Actigraphy was used to evaluate sleep and circadian rhythm, while HRV was monitored for 24 hours to assess autonomic nerve function. Nonparametric indicators were calculated to quantify the active-rest patterns, including interdaily stability, intradaily variability, the most active 10 consecutive hours (M10), the least active 5 consecutive hours (L5), the relative amplitude, and the actigraphic dichotomy index. Follow-ups were conducted at 3 and 6 months after discharge to evaluate prognosis, including the duration of current admission, the number and duration of readmission and ICU admission, and catheterization. Independent sample *t* tests and analysis of covariance were used to compare group differences. Pearson correlation tests were used to explore the correlations of the parameters of actigraphy and HRV with prognosis.

**Results:**

The study included 34 patients with MI (mean age 57.65, SD 9.03 years) and 17 age- and gender-matched controls. MI patients had significantly more wake after sleep onset, an increased number of awakenings, and a lower sleep efficiency than controls. Circadian rhythm analysis revealed significantly lower daytime activity in MI patients. Moreover, these patients had a lower relative amplitude and dichotomy index and a higher intradaily variability and midpoint of M10, suggesting less sleep and wake activity changes, more fragmentation of the rest-activity patterns, and a more delayed circadian rhythm. Furthermore, significant correlations were found between the parameters of circadian rhythm analysis, including nighttime activity, time of M10 and L5, and daytime and nighttime activity_SD_, and patient prognosis.

**Conclusions:**

Patients with acute MI experienced significantly worse sleep and disturbed circadian rhythm compared with healthy controls. Our actigraphy-based analysis revealed a disturbed circadian rhythm, including reduced daytime activities, greater fluctuation in hourly activities, and a weak rest-activity rhythm, which were correlated with prognosis. The evaluation of sleep and circadian rhythm in patients with acute MI can serve as a valuable indicator for prognosis and should be further studied.

## Introduction

Myocardial infarction (MI) is a medical emergency resulting from the occlusion of coronary arteries, which causes impaired perfusion and myocardial damage. It continues to be a leading cause of mortality globally despite progress in clinical management and revascularization techniques, especially in developed countries [1,2]. Annually, more than 2.4 million deaths occur due to MI in the United States, and over 4 million deaths occur across Europe and northern Asia. Furthermore, the burden of cardiovascular disease and MI has increased in middle- and low-income countries, where more than 80% of cardiovascular deaths now occur [3], highlighting its significant socioeconomic impact.

Accumulating evidence shows that the incidence and prognosis of MI, including mortality, cardiovascular events such as reinfarction, admission duration, and readmission, are influenced by numerous factors. Risk factors identified in the INTERHEART study include smoking status, lipid profile, blood pressure, diabetes, obesity, diet, physical activity, alcohol consumption, and psychosocial factors [4,5]. These risk factors account for over 90% of the risk of acute MI and are similar in all geographic regions, races, and genders. According to a previous study [6], when all modifiable risk factors are optimal, the lifetime risk of coronary artery disease or MI for the middle age group is estimated to be less than 5%, and with more than two major risk factors, it rises to 50% for men and 31% for women. These findings highlight the importance of identifying modifiable risk factors. The correlations among sleep, circadian rhythm, and risk of cardiovascular disease have been studied [7,8]. Insomnia has been found to be associated with cardiovascular and metabolic disease risk and mortality [9]. Both short sleep (less than 5 hours) and long sleep (more than 9 hours) can increase the incidence of cardiovascular events [10,11]. Disturbed circadian rhythm due to shift work can also increase cardiovascular risk [12]. Additionally, the frequency and mortality of MI have been found to increase in the morning [13], and patients with onset during the dark-to-light transition period can have a larger infarct size [14]. This is known as the circadian variation of MI, which is related to variations in clock genes [15].

Nowadays, many studies have shown the efficacy of the use of wearable technology in clinical practice and its potential in predicting outcomes. Actigraphy objectively measures patients’ activities and can represent their sleep-wake patterns and sleep quality. Studies have found that monitoring sleep patterns and activity intensity can help in the diagnosis, monitoring, and treatment of depression, anxiety, and narcolepsy [16-18]. Different mathematical algorithm analyses of actigraphy recordings can predict the recurrence of panic attacks in patients with panic disorder and hypersomnia attacks in those with Kleine-Levin syndrome [19,20]. While abundant evidence supports the correlation of heart rate variability (HRV) with the prognosis of MI [21], actigraphy may also provide valuable insights. A study has identified its correlation with the outcomes of patients with heart failure [22], while the study by Peter-Marske et al [23] discovered associations of actigraphy-measured physical activity and sedentary behavior with the incidence of cardiovascular disease, MI, and ischemic stroke. The application of different mathematical algorithms of actigraphy to evaluate sleep and circadian rhythm and their correlations with the prognosis of MI can thus be worthy of investigation.

Currently, most studies have evaluated the premorbid sleep and circadian rhythm of patients with MI and analyzed their correlations. Little is known about sleep and circadian rhythm following the onset of acute MI. Although studies have reported the predictive role of HRV for the prognosis of MI, no study has explored the correlations among sleep, circadian rhythm, and prognosis. Therefore, we conducted a case-control prospective follow-up study to investigate the sleep and circadian rhythm of patients with acute MI, using actigraphy, along with HRV assessment. We followed these patients for 6 months after discharge to evaluate their prognosis. Considering the crucial role of circadian rhythm in cardiovascular function [8], actigraphy data were further analyzed with different mathematical algorithms to assess the circadian rhythm of patients.

## Methods

### Participants

This case-control prospective follow-up study aimed to evaluate sleep and circadian rhythm in patients with acute MI and their correlations with prognosis. Patients who met the inclusion criteria without the exclusion criteria were recruited from the intensive care unit (ICU) following admission. They received standard MI treatments and light therapy during their ICU stay, alongside assessments by a team of cardiologists and psychiatrists experienced in sleep medicine during hospitalization.

The inclusion criteria were as follows: (1) age between 35 and 85 years, (2) diagnosis of acute MI (Killip I-III), and (3) agreement to participate in this study and provide informed consent. The exclusion criteria were as follows: (1) unclear consciousness, (2) use of sedative or hypnotic drugs, (3) blindness or severe cataracts, (4) severe neurological diseases (eg, seizure, brain injury, or severe organic brain diseases), (5) severe psychiatric disorders (eg, schizophrenia, bipolar disorder, mental retardation, or substance use disorder), (6) unwillingness to participate, and (7) high mortality risk.

An age- and gender-matched healthy control group was also recruited for comparison.

### Study Design

**Figure 1:**
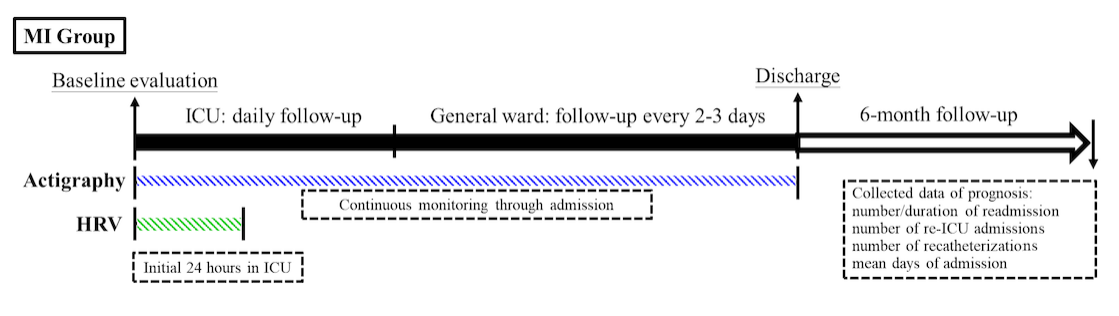
Timeline of the study. Patients with myocardial infarction (MI) underwent actigraphy after admission to the intensive care unit (ICU) and kept wearing the device until discharge. They also underwent heart rate variability (HRV) evaluation for the initial 24 hours in the ICU. After discharge, they were followed for 6 months to evaluate the prognosis.

Upon admission to the ICU, patients were screened and invited if they met the inclusion criteria and were not excluded based on the criteria. Physical evaluation and the diagnosis and treatments of acute MI were conducted by experienced cardiologists, while mental status evaluation was performed by psychiatrists to rule out severe psychiatric disorders. One-hour light therapy was administered each morning during ICU stay to prevent delirium. Baseline evaluation included age, gender, BMI, and blood pressure. Participants also underwent blood sampling for tests, including blood routine tests (white blood cell count, red blood cell count, hemoglobin, hematocrit, mean corpuscular volume, mean corpuscular hemoglobin, mean corpuscular hemoglobin concentration, platelet, platelet distribution width, and mean platelet volume), renal and liver function tests (blood urea nitrogen, creatinine, alanine aminotransferase, and aspartate aminotransferase), electrolyte tests (sodium and potassium), thyroid function tests (thyroid-stimulating hormone and free T4), and vitamin D tests. Actigraphy was used to evaluate sleep and circadian rhythm, while HRV was assessed within 24 hours of ICU admission to evaluate autonomic nerve function (Figure 1). Participants’ conditions were monitored throughout their ICU stay and subsequent general ward stay. Two delirium screening scales (the Confusion Assessment Method for the ICU and the Intensive Care Delirium Screening Checklist) were administered by psychiatrists daily to screen delirium during ICU stay and every 2 days in the general ward [24,25], and only 1 participant had delirium during the study period. Prognosis was evaluated with follow-ups at 3 and 6 months after discharge, and data on prognosis, including the duration of current admission, the number and duration of readmission and ICU admission, and catheterization during the 6-month follow-up period, were collected and confirmed via phone contact and by medical records (Figure 1).

### Actigraphy

**Figure 2:**
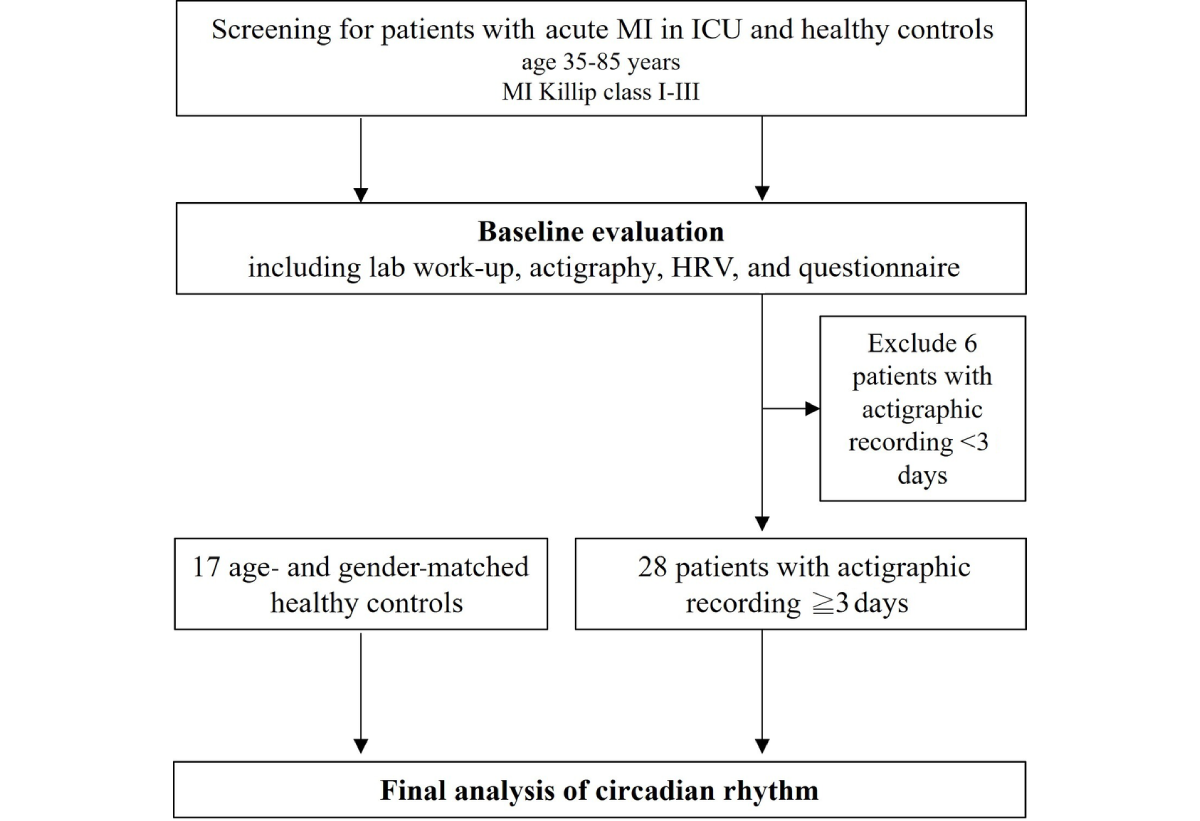
The flowchart outlines the methodological framework for circadian rhythm analysis in the study, from patient recruitment to the processing and analysis of actigraphic data. The chart includes the criteria for patient enrollment based on age and diagnosis, and patients with recording of less than 3 days were excluded from the circadian rhythm analysis. HRV: heart rate variability; ICU: intensive care unit; MI: myocardial infarction.

**Figure 3:**
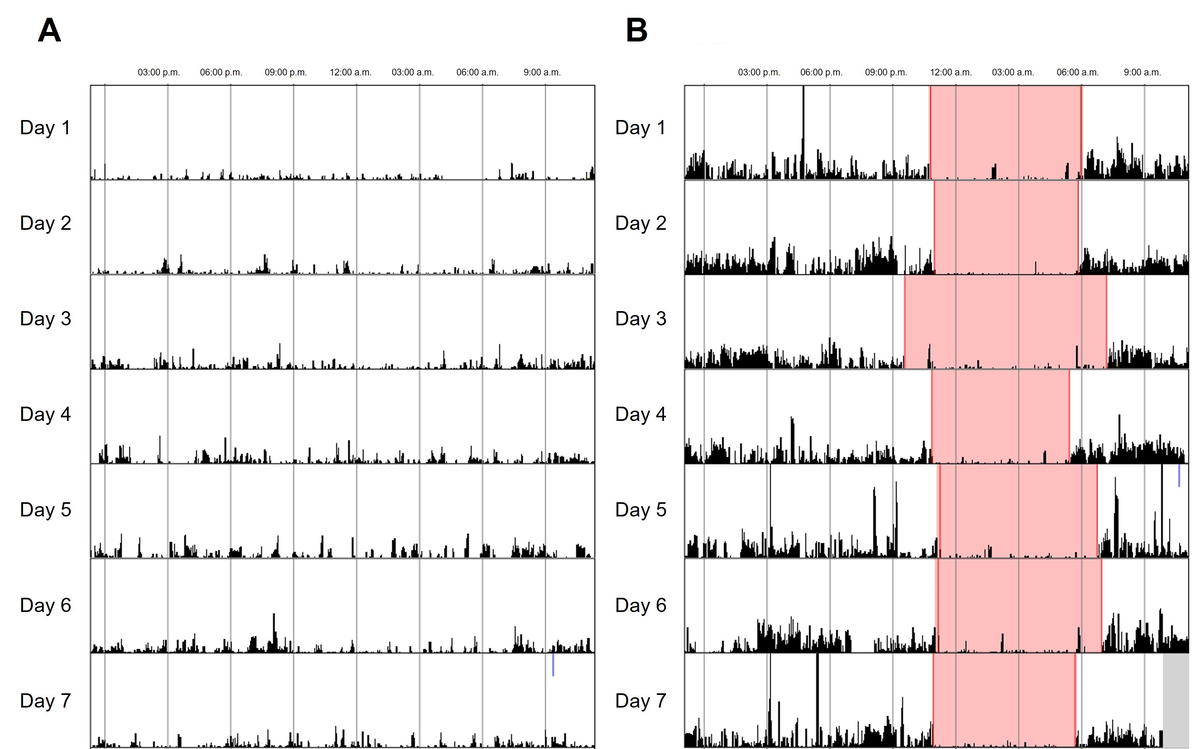
Representative actograms for the myocardial infarction (A) and healthy control (B) groups.

Actigraphy, a nonintrusive approach involving a wristwatch-like device, can monitor participants’ motions and light exposures over extended periods [26]. Data analysis by the widely used parametric method provided sleep parameters, including sleep onset latency, total sleep time, sleep efficiency, wake after sleep onset, and times of awakening, and all 34 participants were included in the analysis. The estimation of these parameters is based on the observation that there is less motion during sleep than during wakefulness [27]. Participants wore the device from enrollment until discharge or withdrawal from the study, which could occur if they met exclusion criteria such as hypnotic use or delirium. Healthy controls wore the device for 2 weeks. To analyze circadian rhythm, participants with actigraphy recording of less than 3 days were excluded. Consequently, 28 of the 34 patients with acute MI and 17 healthy controls were included in the circadian rhythm analysis (Figure 2). Representative actograms for the MI and control groups are shown in Figures 3A and 3B, respectively.

### Circadian Rhythm Analysis and Algorithm of Actigraphy

Since the circadian rhythms of patients with MI can be disrupted and are not easily modeled by a 24-hour sinusoidal wave, we calculated several nonparametric indicators based on the hourly averaged activity from minute-by-minute actigraphy epochs. These indicators are designed to quantify the active-rest patterns and include interdaily stability, intradaily variability, the most active 10 consecutive hours (M10), the least active 5 consecutive hours (L5), the relative amplitude, and the actigraphic dichotomy index (I<O). Detailed mathematical formulas can be found elsewhere [20,28-30]. The interdaily stability and intradaily variability values quantify the stability and fragmentation of the rest-active patterns, respectively, and lower interdaily stability and higher intradaily variability indicate a disrupted circadian rhythm. While M10 and L5 identify the peak and trough of daily activity intensities, relative amplitude quantifies the daily circadian activity amplitude by calculating the difference between M10 and L5, normalized to their means. The midpoints of M10 and L5 represent the central times of these periods. The average daily values of M10, L5, and relative amplitude, and the midpoints of M10 and L5 were used to represent the circadian activity profiles of each participant (Multimedia Appendix 1). The actigraphic dichotomy index (I<O) measures the percentage of in-bed activity counts that fall below the median of out-of-bed activity counts. A lower index suggests a weaker circadian rest-activity rhythm [31].

### HRV Assessment

**Figure 4:**
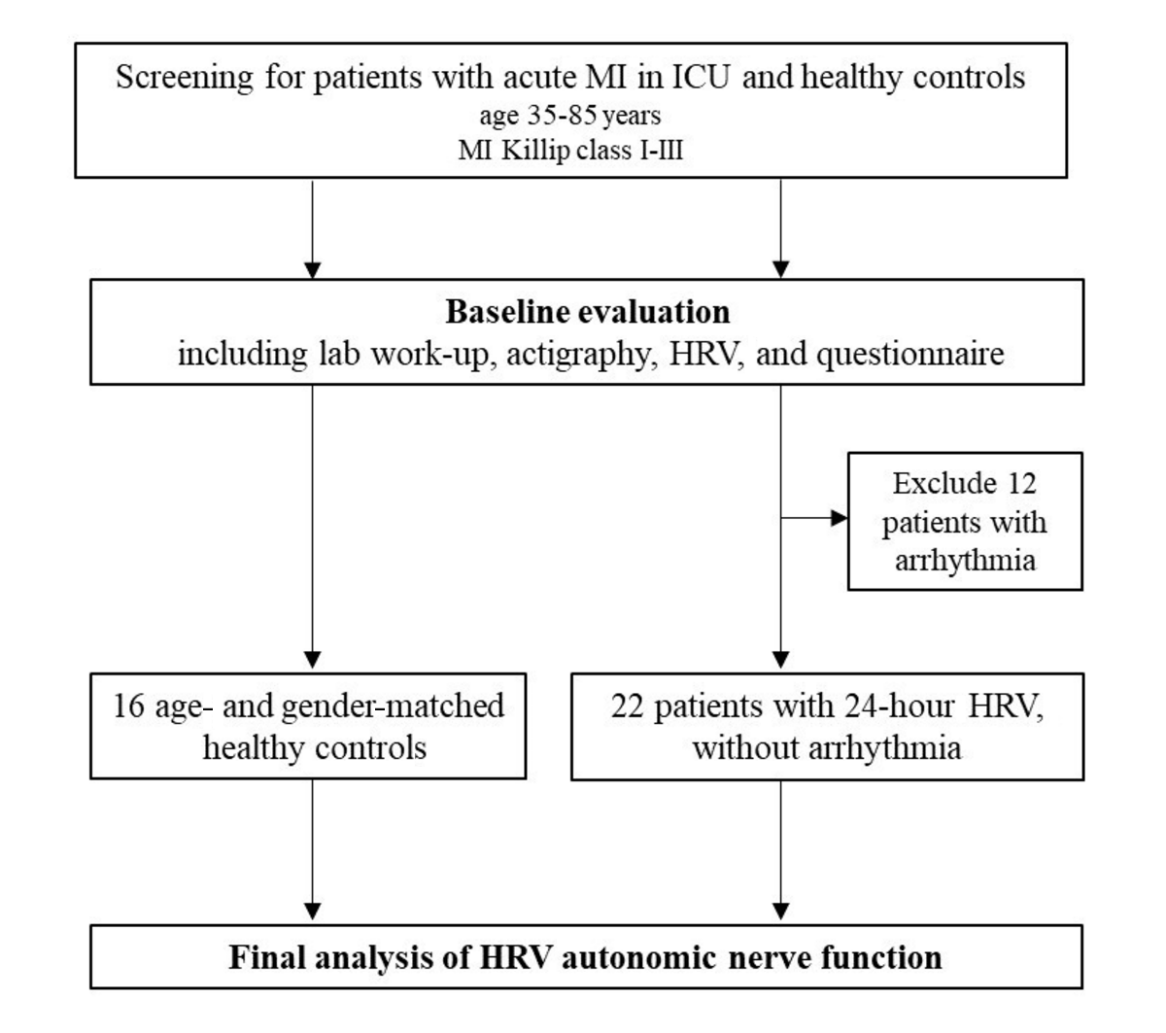
Protocol for heart rate variability (HRV) monitoring within the initial 24 hours of intensive care unit (ICU) admission for patients with acute myocardial infarction (MI). The chart delineates the steps from HRV recording to data analysis.

HRV, reflecting autonomic nervous system tone, was monitored for 24 hours in patients with acute MI after enrollment. Healthy controls underwent HRV assessment for 15 minutes. It has a well-established role as a marker of cardiovascular risk [32]. Previous studies have found that HRV can predict the mortality rate of patients with acute MI [33-35]. Six patients with a history of arrhythmia were excluded, and 22 patients with acute MI and 16 healthy controls were included in the HRV analysis (Figure 4).

### Statistical Analysis

Data were analyzed using SPSS version 20.0 (IBM Corp). Variables were presented by mean and percentage. Independent sample *t* tests and analysis of covariance (ANCOVA) were used to compare differences between the MI group and healthy control group, and age, gender, and BMI were controlled. Pearson correlation tests measured correlations between the parameters of actigraphy and HRV and the prognosis of patients with MI, and partial correlation coefficient (*r*) analysis was used to adjust age and gender. A *P* value of <.05 was considered statistically significant, while a *P* value of <.01 was considered highly statistically significant.

### Ethical Considerations

The study was conducted in accordance with the Declaration of Helsinki and was approved by the Institutional Review Board of Chang Gung Memorial Hospital (201902108A3). Before enrollment, participants were fully informed about the purpose, voluntary participation, and confidentiality of the study, and all signed an informed consent form. Data were anonymized and deidentified. Participants were not offered any form of compensation for their involvement.

## Results

A total of 34 patients with acute MI (mean age 57.65, SD 9.03 years; range 41-73 years; 29 males and 5 females) were enrolled, and all completed the 6-month follow-up. The healthy control group consisted of 17 age- and gender-matched controls (mean age 57.71, SD 12.56 years; 11 males and 6 females). Table 1 shows the comparison of baseline characteristics between the MI group and the healthy control group. BMI was significantly higher in the MI group than in the control group (mean 26.79, SD 4.11 vs mean 23.09, SD 2.15; *P*=.001). After controlling for BMI, laboratory findings showed that many variables were significantly lower in the MI group than in the control group, including vitamin D (mean 17.43, SD 5.00 vs mean 26.53, SD 5.72 ng/mL; *P*<.001), sodium (mean 137.39, SD 2.75 vs mean 139.47, SD 7.25 mEq/L; *P*=.049), potassium (mean 3.83, SD 0.45 vs mean 4.15, SD 0.27 mEq/L; *P*=.04), and thyroid-stimulating hormone levels (mean 0.91, SD 0.46 vs mean 1.66, SD 0.62 μIU/mL; *P*=.02). On the other hand, some variables were significantly higher in the MI group than in the control group, including white blood cell count (mean 9.87, SD 2.56 vs mean 6.47, SD 1.87 ×1000/μL; *P*<.001), aspartate aminotransferase (mean 37.06, SD 23.77 vs mean 19.53, SD 3.47 U/L; *P*=.02), and alanine aminotransferase (mean 31.88, SD 17.62 vs mean 19.71, SD 5.72 U/L; *P*=.04). Of the 34 participants, 5 (18%) experienced initial symptoms of MI within an hour after awakening, and the onset time for 6 participants could not be confirmed due to atypical clinical presentations.

**Table 1:** Comparison of baseline characteristics between the myocardial infarction and healthy control groups.

Variable			Myocardial infarction group (n=34)		Healthy control group (n=17)		*P* value^a^
Age (years), mean (SD)			57.65 (9.03)		57.71 (12.56)		.99
**Gender, n (%)**							.09
	Male	29 (85)		11 (65)			
	Female	5 (15)		6 (35)			
BMI (kg/m^2^), mean (SD)			26.79 (4.11)		23.09 (2.15)		.001
Initial symptoms within 1 hour after awakening, n (%)			5 (18)		—^b^		—
**Laboratory finding, mean (SD)**							
	Total vitamin D (ng/mL)	17.43 (5.00)		26.53 (5.72)		<.001	
	WBC^c^ (1000/μL)	9.87 (2.56)		6.47 (1.87)		<.001	
	RBC^d^ (million/μL)	4.64 (1.01)		4.74 (0.20)		.42	
	HGB^e^ (g/dL)	13.89 (2.87)		14.16 (0.74)		.64	
	HCT^f^ (%)	41.56 (8.33)		43.09 (2.07)		.37	
	MCV^g^ (fL)	90.04 (6.48)		90.91 (2.36)		.79	
	MCH^h^ (pg/cell)	30.11 (2.63)		29.88 (0.86)		.22	
	MCHC^i^ (gHb/dL)	33.40 (1.38)		32.89 (0.90)		.07	
	RDW^j^ (%)	13.26 (1.49)		12.77 (0.64)		.76	
	Platelet (1000/μL)	237.00 (64.28)		215.82 (45.94)		.90	
	PDW^k^ (fL)	10.44 (1.60)		9.91 (1.58)		.53	
	MPV^l^ (fL)	9.72 (0.73)		9.39 (0.81)		.31	
	BUN^m^ (mg/dL)	31.79 (33.32)		16.25 (4.20)		.19	
	Creatinine (mg/dL)	2.66 (3.95)		0.81 (0.22)		.19	
	ALT^n^ (U/L)	31.88 (17.62)		19.71 (5.72)		.04	
	AST^o^ (U/L)	37.06 (23.77)		19.53 (3.47)		.02	
	Na (mEq/L)	137.39 (2.75)		139.47 (7.25)		.049	
	K (mEq/L)	3.83 (0.45)		4.15 (0.27)		.04	
	Free T4 (ng/dL)	0.98 (0.22)		0.93 (0.11)		.97	
	TSH^p^ (μIU/mL)	0.91 (0.46)		1.66 (0.62)		.002	

^a^*P* values were calculated using independent sample *t* tests; laboratory findings were assessed using analysis of covariance (ANCOVA) controlling for BMI.

^b^Not applicable.

^c^WBC: white blood cell.

^d^RBC: red blood cell.

^e^HGB: hemoglobin.

^f^HCT: hematocrit.

^g^MCV: mean corpuscular volume.

^h^MCH: mean corpuscular hemoglobin.

^i^MCHC: mean corpuscular hemoglobin concentration.

^j^RDW: red cell distribution width.

^k^PDW: platelet distribution width.

^l^MPV: mean platelet volume.

^m^BUN: blood urea nitrogen.

^n^ALT: alanine aminotransferase.

^o^AST: aspartate aminotransferase.

^p^TSH: thyroid-stimulating hormone.

**Figure 5:**
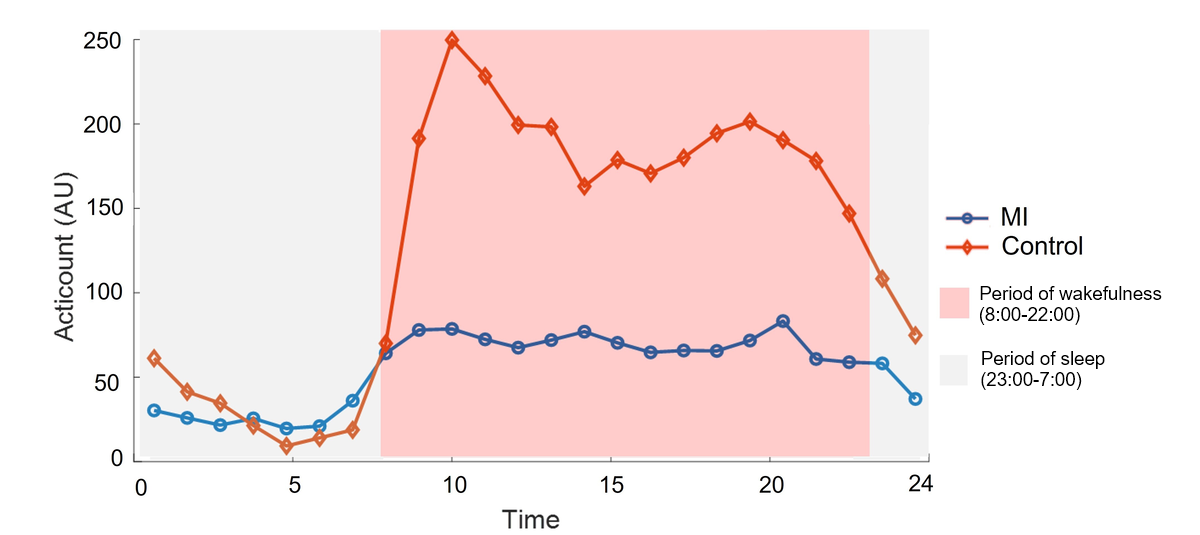
The average circadian activity template of the myocardial infarction (MI) patient cohort, synthesized from actigraphy data. It depicts the group’s collective rest-activity cycles over a standard 24-hour period, highlighting the marked decreased amplitude of daytime and nighttime activity changes observed after acute MI.

**Figure 6:**
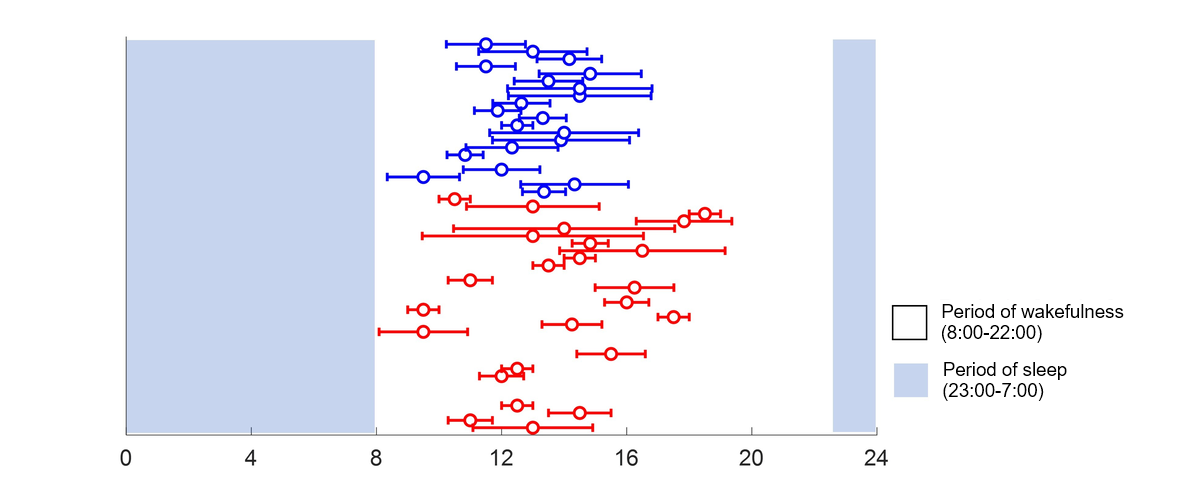
Acrophase map showing the timings of the midpoint of M10 of myocardial infarction (MI) patients and healthy controls. Patients with MI had higher individual variability in the timing of the midpoint of M10 compared to healthy controls. M10: most active 10 consecutive hours.

Table 2 shows the comparison of the general parametric sleep parameters of actigraphy between the MI group and the healthy control group after controlling for age, gender, and BMI. In the MI group, the average duration of actigraphy monitoring was 5.75 (SD 2.01) days. The parametric analysis of actigraphy showed that patients with MI had significantly more wake after sleep onset (mean 97.70, SD 59.95 vs mean 50.2, SD 18.17 min; *P*=.01), increased number of awakenings (mean 33.29, SD 12.61 vs mean 22.25, SD 6.14 times; *P*=.007), and lower sleep efficiency (mean 77.96, SD 7.73% vs mean 84.65, SD 4.03%; *P*=.01) than controls (Table 2). Circadian rhythm analysis of actigraphy is shown in Table 3 after controlling for age, gender, and BMI. Compared with the control group, the circadian patterns in the MI group showed less diurnal change and more individual differences (Figures 5 and 6; Multimedia Appendix 2 and 3). Moreover, patients with MI had significantly lower daytime activity (mean 70.65, SD 32.74 vs mean 188.46, SD 84.53; *P*<.001) and averaged M10 (mean 91.00, SD 42.79 vs mean 233.66, SD 106.05; *P*<.001) than controls, suggesting lower activity when awake. Both nighttime activity and averaged L5 were not significantly different between the groups, but the MI group had significantly lower daytime (mean 130.27, SD 39.55 vs mean 283.69, SD 100.78; *P*<.001) and nighttime activity_SD_ (mean 87.13, SD 42.12 vs mean 122.58, SD 43.71; *P*=.03), suggesting less daytime and nighttime activity daily changes. The MI group had a significantly lower relative amplitude (mean 0.69, SD 0.13 vs mean 0.87, SD 0.10; *P*<.001) and dichotomy index (mean 0.85, SD 0.05 vs mean 0.93, SD 0.04; *P*<.001) than the control group, indicating that patients with MI had less sleep and wake activity changes and a weak active-rest rhythm. Moreover, the significantly higher intradaily variability in the MI group than in the control group (mean 1.13, SD 0.27 vs mean 0.94, SD 0.21; *P*=.02) suggested that the MI group had more fragmentations and disturbances during the wake and sleep states. The time of M10 (mean 14.86, SD 1.76 vs mean 13.52, SD 1.07; *P*=.02) was significantly higher in the MI group than in the control group, which supported a more delayed circadian rhythm.

**Table 2:** Comparison of the results of actigraphy between the myocardial infarction and healthy control groups after controlling for age, gender, and BMI.

Variable	Myocardial infarction group (n=28)	Healthy control group (n=17)	*P* value^a^
SOL^b^ (min), mean (SD)	33.99 (34.65)	14.40 (9.98)	.11
TST^c^ (min), mean (SD)	501.74 (199.41)	420.59 (88.32)	.15
SE^d^ (%), mean (SD)	77.96 (7.73)	84.65 (4.03)	.01
WASO^e^ (min), mean (SD)	97.70 (59.95)	50.2 (18.17)	.01
Awake (times), mean (SD)	33.29 (12.61)	22.25 (6.14)	.007

^a^*P* values were calculated using analysis of covariance (ANCOVA) controlling for age, gender, and BMI.

^b^SOL: sleep onset latency.

^c^TST: total sleep time.

^d^SE: sleep efficiency.

^e^WASO: wake after sleep onset.

**Table 3:** Comparison of the results of circadian rhythm analysis of actigraphy between the myocardial infarction and healthy control groups after controlling for age, gender, and BMI.

Variable			Myocardial infarction group (n=28)		Healthy control group (n=17)		*P* value^a^
**Activity count, mean (SD)**							
	Daytime activity	70.65 (32.74)		188.46 (84.53)		<.001	
	Daytime activity_SD_	130.27 (39.55)		283.69 (100.78)		<.001	
	Nighttime activity	33.16 (26.65)		37.45 (14.86)		.53	
	Nighttime activity_SD_	87.13 (42.12)		122.58 (43.71)		.03	
**Active-rest rhythm, mean (SD)**							
	M10^b^	91.00 (42.79)		233.66 (106.05)		<.001	
	Time of M10	14.86 (1.76)		13.52 (1.07)		.02	
	L5^c^	16.02 (9.10)		14.84 (8.38)		.92	
	Time of L5	6.47 (7.73)		2.80 (1.25)		.08	
	RA^d^	0.69 (0.13)		0.87 (0.10)		.001	
	IV^e^	1.13 (0.27)		0.94 (0.21)		.02	
	IS^f^	0.31 (0.12)		0.41 (0.12)		.06	
	Dichotomy index	0.85 (0.05)		0.93 (0.04)		<.001	

^a^*P* values were calculated using analysis of covariance (ANCOVA) controlling for age, gender, and BMI.

^b^M10: most active 10 consecutive hours.

^c^L5: least active 5 consecutive hours.

^d^RA: relative amplitude.

^e^IV: intradaily variability.

^f^IS: interdaily stability.

Table 4 shows the comparison of the parameters of HRV between the MI group and the healthy control group after controlling for age, gender, and BMI. The MI group showed a significantly lower root mean square of successive differences (mean 17.27, SD 9.49 vs mean 28.94, SD 17.22; *P*=.04), percentage of successive RR intervals that differ by more than 50 ms (mean 3.19, SD 4.63 vs mean 12.33, SD 16.60; *P*=.04), Poincaré plot SD perpendicular to the line of identity (SD1) (mean 12.20, SD 6.70 vs mean 20.43, SD 12.16; *P*=.04), and SD ratio (mean 0.27, SD 0.09 vs mean 0.44, SD 0.23; *P*=.01) than the control group, suggesting lower parasympathetic nerve function in the MI group.

**Table 4:** Comparison of the results of heart rate variability between the myocardial infarction and healthy control groups after controlling for age, gender, and BMI.

Variable	Myocardial infarction group (n=22)	Healthy control group (n=16)	*P* value^a^
Overall, mean (SD)	836.35 (129.88)	878.91 (130.74)	.40
HR^b^, mean (SD)	73.53 (10.12)	69.65 (9.96)	.32
SDRR^c^, mean (SD)	37.89 (19.59)	37.38 (15.39)	.90
RMSSD^d^, mean (SD)	17.27 (9.49)	28.94 (17.22)	.04
pNN50^e^, mean (SD)	3.19 (4.63)	12.33 (16.60)	.04
pNN20^f^, mean (SD)	21.45 (19.14)	38.34 (25.12)	.13
SD1^g^, mean (SD)	12.20 (6.70)	20.43 (12.16)	.04
SD2^h^, mean (SD)	51.83 (27.08)	47.96 (19.96)	.84
SD ratio^i^, mean (SD)	0.27 (0.09)	0.44 (0.23)	.01
DC value^j^, mean (SD)	6.25 (3.33)	9.22 (5.40)	.25

^a^*P* values were calculated using analysis of covariance (ANCOVA) controlling for age, gender, and BMI.

^b^HR: heart rate.

^c^SDRR: SD of the RR interval.

^d^RMSSD: root mean square of successive differences.

^e^pNN50: percentage of successive RR intervals that differ by more than 50 ms.

^f^pNN20: percentage of successive RR intervals that differ by more than 20 ms.

^g^SD1: Poincaré plot SD perpendicular to the line of identity.

^h^SD2: Poincaré plot SD along the line of identity.

^i^SD ratio: ratio of SD1 to SD2.

^j^DC value: deceleration capacity of heart rate.

Table 5 shows the correlations between the parameters of actigraphy and the prognosis of patients with MI during the 6-month follow-up. Significantly positive correlations were found between nighttime activity and total days of current admission (*r*=0.529; *P*=.004); between time of M10 and any ICU readmission (*r*=0.452; *P*=.02), times of readmission (*r*=0.496; *P*=.007), total days of readmission (*r*=0.571; *P*=.001), and mean days of readmission (*r*=0.459; *P*=.01); and between time of L5 and total days of current admission (*r*=0.504; *P*=.006). Significantly negative correlations were found between daytime activity_SD_ and any ICU readmission (*r*=–0.417; *P*=.03), times of readmission (*r*=–0.415; *P*=.03), and total days of readmission (*r*=–0.450; *P*=.02); and between nighttime activity_SD_ and times of readmission (*r*=–0.421; *P*=.03).

**Table 5:** Correlations (r) of the parameters of actigraphy and the prognosis of patients with myocardial infarction (n=28).

Variable		Total days of current admission	Any readmission	Any ICU^a^ readmission	Times of readmission	Total days of readmission	Any catheterization	Mean days of readmission
TST^b^		–0.164	0.166	0.092	0.199	0.066	0.309	–0.011
SOL^c^		–0.089	0.068	–0.172	0.044	–0.111	0.283	–0.134
SE^d^		0.141	0.305	0.158	0.310	0.281	0.228	0.303
WASO^e^		–0.075	0.260	–0.039	0.204	–0.065	0.279	–0.068
Awake number		–0.304	0.120	0.104	0.120	–0.122	0.207	–0.206
**Activity counts**								
	Daytime activity	0.347	–0.107	–0.334	–0.269	–0.314	–0.265	–0.152
	Daytime activity_SD_	0.171	–0.269	–0.417^f^	–0.415^f^	–0.450^f^	–0.271	–0.330
	Nighttime activity	0.529^g^	–0.068	–0.151	–0.171	–0.138	–0.166	0.006
	Nighttime activity_SD_	0.314	–0.319	–0.357	–0.421^f^	–0.358	–0.288	–0.246
**Active-rest rhythm**								
	M10^h^	0.369	–0.176	–0.351	–0.326	–0.336	–0.312	–0.180
	Time of M10 (pm)	–0.040	0.358	0.452^f^	0.496^g^	0.571^g^	0.125	0.459^f^
	L5^i^	0.352	–0.117	–0.256	–0.255	–0.317	–0.100	–0.206
	Time of L5 (am)	0.504^g^	0.207	0.088	0.079	–0.002	0.004	0.167
	RA^j^	–0.074	–0.130	–0.097	–0.107	–0.028	–0.169	–0.012
	IV^k^	–0.124	0.282	0.328	0.343	0.315	0.122	0.250
	IS^l^	–0.332	–0.060	–0.227	–0.153	–0.312	–0.050	–0.293
	Dichotomy index	–0.354	–0.143	0.029	–0.016	0.101	–0.261	–0.011

^a^ICU: intensive care unit.

^b^TST: total sleep time.

^c^SOL: sleep onset latency.

^d^SE: sleep efficiency.

^e^WASO: wake after sleep onset.

^f^*P*<.05.

^g^*P*<.01.

^h^M10: most active 10 consecutive hours.

^i^L5: least active 5 consecutive hours.

^j^RA: relative amplitude.

^k^IV: intradaily variability.

^l^IS: interdaily stability.

After adjusting for age and gender, there were still significant correlations between several parameters of actigraphy and the prognosis of patients with MI (Multimedia Appendix 4). Significantly positive correlations were found between nighttime activity and total days of current admission (*r*=0.56; *P*=.003); and between time of M10 and any ICU readmission (*r*=0.457; *P*=.02), times of readmission (*r*=0.527; *P*=.006), total days of readmission (*r*=0.579; *P*=.002), and mean days of readmission (*r*=0.466; *P*=.02). Significantly negative correlations were found between IS and recatheterization (*r*=–0.423; *P*=.03) and mean days of readmission (*r*=–0.413; *P*=.04).

Table 6 shows the correlations between the parameters of HRV and the prognosis of patients with MI during the 6-month follow-up. Significantly positive correlations were found between the SD ratio and days of readmission (*r*=0.425; *P*=.049), recatheterization (*r*=0.426; *P*=.048), and mean days of readmission (*r*=0.425; *P*=.049). Significantly negative correlations were found between the SD of the RR interval and any readmission (*r*=–0.447; *P*=.04) and times of readmission (*r*=–0.447; *P*=.04); between Poincaré plot SD along the line of identity (SD2) and any readmission (*r*=–0.448; *P*=.04), times of readmission (*r*=–0.448; *P*=.04), days of readmission (*r*=–0.424; *P*=.049), and mean days of readmission (*r*=–0.424; *P*=.049); and between the deceleration capacity of heart rate (DC value) and any readmission (*r*=–0.457; *P*=.03) and times of readmission (*r*=–0.457; *P*=.03).

**Table 6:** Correlations (r) of the parameters of heart rate variability and the prognosis of patients with myocardial infarction (n=22).

Variable	Total days of current admission	Any readmission	Any ICU^a^ readmission	Times of readmission	Total days of readmission	Any catheterization	Mean days of readmission
Overall mean	0.053	–0.260	–0.069	–0.260	–0.173	–0.121	–0.173
HR^b^	–0.072	0.245	0.041	0.245	0.147	0.096	0.147
SDRR^c^	0.189	–0.447^d^	–0.032	–0.447^d^	–0.421	–0.314	–0.421
RMSSD^e^	–0.038	–0.370	–0.159	–0.370	–0.323	–0.164	–0.323
pNN50^f^	0.062	–0.339	–0.142	–0.339	–0.301	–0.175	–0.301
pNN20^g^	–0.037	–0.351	–0.182	–0.351	–0.317	–0.100	–0.317
SD1^h^	–0.038	–0.370	–0.159	–0.370	–0.323	–0.164	–0.323
SD2^i^	0.199	–0.448^d^	–0.023	–0.448^d^	–0.424^d^	–0.321	–0.424^d^
SD ratio^j^	–0.116	0.317	–0.294	0.317	0.425^d^	0.426^d^	0.425^d^
DC value^k^	–0.095	–0.457^d^	–0.126	–0.457^d^	–0.403	–0.278	–0.403

^a^ICU: intensive care unit.

^b^HR: heart rate.

^c^SDRR: SD of the RR interval.

^d^*P*<.05.

^e^RMSSD: root mean square of successive differences.

^f^pNN50: percentage of successive RR intervals that differ by more than 50 ms.

^g^pNN20: percentage of successive RR intervals that differ by more than 20 ms.

^h^SD1: Poincaré plot SD perpendicular to the line of identity.

^i^SD2: Poincaré plot SD along the line of identity.

^j^SD ratio: ratio of SD1 to SD2.

^k^DC value: deceleration capacity of heart rate.

After adjusting for age and gender, there were fewer significant correlations between the parameters of HRV and the prognosis of patients with MI (Multimedia Appendix 5). Significantly positive correlations were found only between the SD ratio and days of readmission (*r*=0.455; *P*=.04), recatheterization (*r*=0.456; *P*=.04), and mean days of readmission (*r*=0.455; *P*=.04).

## Discussion

### Principal Findings

Previous studies have used actigraphy to evaluate activities and sleep in patients with MI [23,36,37]. However, to our knowledge, no study has examined the circadian rhythm of patients after the onset of acute MI using actigraphy and analyzed its correlations with prognosis. Therefore, our findings are significant as they not only reveal sleep and circadian rhythm disturbances after acute MI but also support the application of actigraphy and HRV in evaluating the prognosis.

Unsurprisingly, baseline laboratory work-up assessments showed significant differences between patients with MI and healthy controls. Patients with MI exhibited more obesity and more abnormalities, including a lower vitamin D level, which is known to be negatively associated with the incidence of cardiovascular diseases [38,39]. This study further revealed significant differences in sleep patterns between the 2 groups. After acute MI, patients had significantly worse sleep, characterized by more wake after sleep onset and number of awakenings, and lower sleep efficiency. These findings align with a previous study, which has reported sleep disturbances in patients after acute MI, including more wakefulness and awakening, more stage shifts, less rapid eye movement, decreased sleep efficiency, and increased daytime sleep [40]. These changes can be attributed to MI itself rather than differences in a hospital setting [40] and can be normalized with an improved physical condition. Sleep has been proposed as a vital sign and a marker for disease and recovery [41], and thus, routine monitoring and evaluation of sleep are recommended in patients with MI.

The circadian rhythm analysis using actigraphy data supports that both sleep quality and circadian rhythm can be disturbed in patients with MI. Compared with controls, the activities of patients with MI were significantly less during the daytime, while nighttime activities remained unchanged. Moreover, there was less variability (SD) in both daytime and nighttime activities, indicating less change in activity levels throughout the day. Although these findings may be linked to the bed rest and activity restrictions commonly advised for patients with acute MI, the higher intradaily variability contradicts this hypothesis and instead suggests greater fluctuations in hourly activities during both the daytime and nighttime. Furthermore, a lower relative amplitude and actigraphic dichotomy index suggested less distinct activity changes between sleep and wakefulness and a weak circadian rest-activity rhythm. Patients with MI also exhibited a significant delay in their circadian rhythm, as indicated by a higher time of M10.

Most studies on circadian rhythm and MI have focused on the circadian variances of MI [42-44], which can be explained by the fact that circadian rhythm–related coronary vasoconstriction and increased sympathetic tone may disrupt atherosclerotic plaque [45]. In this study, 5 out of 34 participants (18%) experienced initial symptoms within 1 hour after awakening, but we did not find any correlation between their onset time and prognosis. The nonsignificant result can be influenced by several factors. We only enrolled patients with Killip I to III and excluded those with severe comorbidities and a high mortality risk. Some participants could have recall bias due to their atypical clinical presentations. Circadian variances of MI and their correlations with prognosis necessitate further investigation.

Delayed and disturbed circadian rhythm has been found to be associated with cardiovascular diseases [46,47]. Consistent with our findings, a cross-sectional study also found a lower 24-hour rest-activity rhythm amplitude and overall rhythmicity in patients with coronary artery disease, using actigraphy [48]. Although little is known about circadian rhythm after the onset of MI, our findings indicate that several parameters derived from the circadian rhythm analysis have significant correlations with prognosis, including the total days of current admission, ICU readmission, and the number and duration of readmissions during the 6-month follow-up period. Patients with a more delayed circadian rhythm (higher times of L5 and M10) were more likely to have a longer duration of current admission, more frequent readmissions, and a longer duration of readmission. Since gender and age can have impacts on both sleep and the prognosis of MI [49-51], we adjusted age and gender in the correlation analysis. Most of the significant correlations could still be found (Multimedia Appendix 4). Although a causal relationship cannot be confirmed, these findings are consistent with those of previous studies that suggest abnormal circadian rhythm as a core vulnerability factor for MI [15,52]. Interventions targeting the circadian rhythm of patients with MI, especially those with a disturbed and delayed sleep phase, may be beneficial. Exercise programs and rehabilitations have already been demonstrated to improve prognosis and reduce mortality in patients with MI [53], and they can also help improve sleep. During the acute phase, light therapy and environmental modifications may aid in the regulation of circadian rhythm and are worthy of further investigation.

We found that patients with MI had lower parasympathetic activities compared to healthy controls, and there were correlations between several HRV parameters and prognosis. HRV is an indirect measure of autonomic nervous activity [29] and has been investigated for its ability to predict prognosis in patients with acute MI. Previous studies have reported associations between HRV and mortality or cardiovascular events [54-57]. In this study, better parasympathetic nerve activity after acute MI could be correlated with a better prognosis. However, we also found positive correlations between the ratio of SD1 to SD2 (SD ratio) and prognosis, including recatheterization and the duration of readmission, contradicting other findings. A previous study found a correlation between a high SD ratio and arrhythmia such as ventricular tachycardia [58]. Thus, although a higher parasympathetic nerve activity can correlate with a better prognosis, the SD ratio may not always be “the higher, the better.”

Comparing correlations with the prognosis of MI, the circadian rhythm analysis using actigraphy showed more significant findings than the HRV analysis, especially in nighttime activity and the time of M10. Moreover, fewer significant correlations between HRV parameters and prognosis were found after adjusting for age and gender (Multimedia Appendix 5). These findings suggest that the evaluation of sleep and circadian rhythm after the onset of MI is a valuable prognosis indicator. Previous studies have demonstrated the potential of actigraphy to predict the outcomes of various diseases [19,20]. The application of actigraphy in the evaluation and treatment of patients with MI is promising, as it has already been suggested to play a role in the prognostication of heart failure [59]. Another study showed that the evaluation of subjective sleep complaints through questionnaires may serve as a prognostic marker in patients after acute MI [60], emphasizing the importance of sleep in the risk stratification of MI. Although our study found fewer HRV correlations with prognostic factors, it differed from past research as there was no morality after excluding patients with Killip IV. Furthermore, instead of analyzing mortality, our study focused on correlations with prognostic factors such as admission duration, readmission, and recatheterization. Given that only 22 cases were included in the HRV analysis, factors, including age, gender, and medications such as beta-blockers [61], might have affected the findings. Further research is required to compare the 2 evaluation tools in terms of their correlations with the prognosis of MI.

### Limitations

The study has some limitations. First, the study had a small sample size, which limits detailed subgroup analysis by age or gender. Second, we only evaluated participants after the onset of MI during their admission, and therefore, the observed delayed and disturbed circadian rhythm may not represent their premorbid sleep and circadian rhythm. They did not undergo actigraphy monitoring during follow-up, and thus, subsequent changes in sleep and circadian rhythm were not known. Further studies following MI patients with actigraphy after discharge can increase the understanding of the long-term changes in sleep and circadian rhythm after MI and their impacts on prognosis. Third, patients in our study received light therapy during their stay in the ICU to prevent delirium. However, the duration of light therapy was short (1 hour per day for only 1-2 days), limiting its potential impacts. Fourth, some patients with MI were excluded, such as those classified as Killip IV. Therefore, caution is required when generalizing our findings, especially to severe cases with high mortality rates. Lastly, comorbidities that can contribute to sleep disturbance were not controlled. Sleep disorders, such as obstructive sleep apnea, cannot be ruled out, as polysomnography is challenging to perform in patients with acute MI in the ICU setting. Nevertheless, our findings not only support but also extend the use of actigraphy beyond its conventional role in sleep medicine to include applications within cardiology.

### Conclusions

Patients with acute MI experienced significantly worse sleep and disturbed circadian rhythm compared with healthy controls. Actigraphy-based analysis revealed a disturbed circadian rhythm, including reduced daytime activities, a greater fluctuation in hourly activities, and a weak rest-activity rhythm, which were correlated with prognosis. The evaluation of sleep and circadian rhythm in patients with acute MI can serve as a valuable indicator for prognosis and warrants further investigation.

## Appendix

Multimedia Appendix 1 Circadian rhythm analysis.

Multimedia Appendix 2 Individual circadian rhythm profiles of patients with myocardial infarction (MI).

Multimedia Appendix 3 Individual circadian rhythm profiles of healthy controls.

Multimedia Appendix 4 Correlations of the parameters of actigraphy and prognosis of patients with myocardial infarction after adjusting for age and gender.

Multimedia Appendix 5 Correlations of the parameters of heart rate variability and prognosis of patients with myocardial infarction after adjusting for age and gender.

## Abbreviations

HRV: heart rate variability
ICU: intensive care unit
L5: least active 5 consecutive hours
M10: most active 10 consecutive hours
MI: myocardial infarction
SD1: Poincaré plot SD perpendicular to the line of identity
SD2: Poincaré plot SD along the line of identity

## Data Availability

Anonymized data that support the findings of this study are available upon reasonable request from the corresponding author (YSH).
