# Supplementary material for: The Prognostic Significance of Sleep and Circadian Rhythm for Myocardial Infarction Outcomes: Case-Control Study
Source: J Med Internet Res. 2025 Feb 4;27:e63897. doi: 10.2196/63897 (PMC11836589; doi:10.2196/63897)
Supplement: Multimedia Appendix 1 [file jmir_v27i1e63897_app1.docx]

## **Multimedia Appendix 1.** Circadian rhythm analysis.

### 1. Analysis of Circadian Rhythm Analysis with Actigraphy Data

Our analysis of circadian rhythm analysis utilized MATLAB software (MathWorks, Natick, USA) and incorporated established algorithms including the cosinor model and nonparametric parameters. Detailed description of the algorithms can be found elsewhere [1,2]. Initially, minute-by-minute acticounts were aggregated and averaged hourly, and circadian patterns of these activity data were assessed through parametric and nonparametric analyses. For parametric analysis, we employed a cosinor model featuring a 24-hour sinusoidal function, defined by:

$$A\left( t \right)=M+A\cos\left( \frac{2\pi t}{24}-\varphi\right)+\varepsilon$$

M denotes the midline estimating statistic of rhythm (MESOR), $\varphi$ represents the acrophase indicating the time of peak activity in a 24-hour rhythm, and $\varepsilon$ denotes the unexplained residual. The coefficient A indicates the amplitude of the fitted cosine function.

Relative amplitude (RA), a nonparametric circadian variable, was analyzed by the formula:

$$RA=\frac{M10-L5}{M10+L5}$$

M10 is defined by the most active 10 consecutive hours, and L5 the least active five consecutive hours, which are utilized for estimating daily peak and trough of activity. Daily RA quantifies the amplitude from peak to trough, normalized to the mean activity. Overall RA was derived from averaging daily RA values. Additionally, midpoints of M10 and L5 were determined by averaging the daily time points in the middle of M10 and L5 [1,2].

### 2. Assessing Rest-Activity Patterns with Actigraphy Data

Two nonparametric variables, interdaily stability (IS) and intradaily variability (IV), were employed to quantify the stability and the fragmentation of rest-activity rhythm respectively. IS and IV are analyzed by the following formulas:

$$IS=\frac{\frac{\sum_{h=1}^{p} {(x_{h}-\bar{x})}^{2}}{p}}{\frac{\sum_{i=1}^{N} {(x_{i}-\bar{x})}^{2}}{N}}$$

N is the total hourly activity, x_i_ is the sequence of activity points, p is the number of data sets per day, x_h_ is the average of the activity of a specific 24-hour time, and x̄ represents the mean of all activity.

$$IV=\frac{\frac{\sum_{i=1}^{N-1} {(x_{i+1}-x_{i})}^{2}}{N-1}}{\frac{\sum_{i=1}^{N} {(x_{i}-\bar{x})}^{2}}{N}}$$

N, x_i_, and x̄ are as previously defined. IV is calculated on an hourly basis by summing the differences in hourly activity between successive hours and normalized by the overall activity variance. Higher IS values suggest more regular activity patterns, and lower IV values indicate less fragmented rhythm, that is, less fluctuation between active and rest patterns.

## References

1. Lin C, Chin WC, Huang YS, Chu KC, Paiva T, Chen CC and Guilleminault C. Different circadian rest-active rhythms in Kleine-Levin syndrome: a prospective and case-control study. Sleep 2021;44(9). doi:0.1093/sleep/zsab096
2. Yang, HW, Garaulet M, Li P, Bandin C, Lin C, Lo MT, Hu K. Daily Rhythm of Fractal Cardiac Dynamics Links to Weight Loss Resistance: Interaction with CLOCK 3111T/C Genetic Variant. Nutrients 2021;13(7). doi:10.3390/nu13072463
