# Supplementary material for: The Prognostic Significance of Sleep and Circadian Rhythm for Myocardial Infarction Outcomes: Case-Control Study
Source: J Med Internet Res. 2025 Feb 4;27:e63897. doi: 10.2196/63897 (PMC11836589; doi:10.2196/63897)

**Multimedia Appendix 2.** Individual circadian rhythm profiles of patients with myocardial infarction (MI). This figure contrasts the daily activity patterns, capturing the variability and disruptions in their active-rest cycles. It visually summarizes how each patient’s circadian rhythm deviates from the norm following an acute MI.
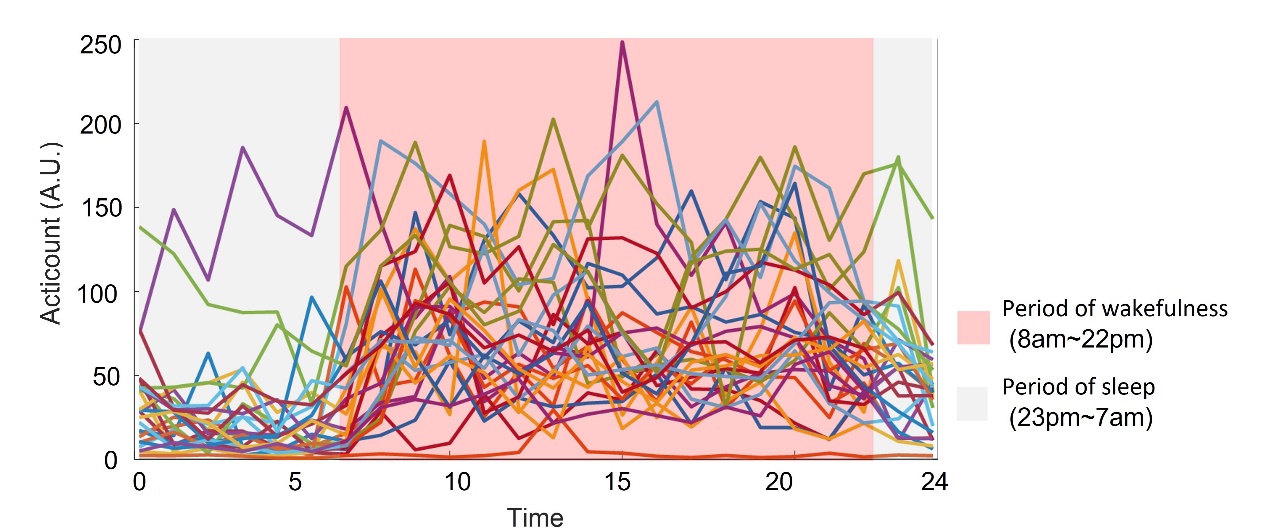

Supplement: Multimedia Appendix 2 [file jmir_v27i1e63897_app2.docx]
