# Supplementary material for: The Prognostic Significance of Sleep and Circadian Rhythm for Myocardial Infarction Outcomes: Case-Control Study
Source: J Med Internet Res. 2025 Feb 4;27:e63897. doi: 10.2196/63897 (PMC11836589; doi:10.2196/63897)

**Multimedia Appendix 3.** Individual circadian rhythm profiles of healthy controls. It depicts the normal variability in daily activities. It allows for a direct comparison with the MI patients' profiles, accentuating the discrepancies in circadian patterns due to the impact of MI.
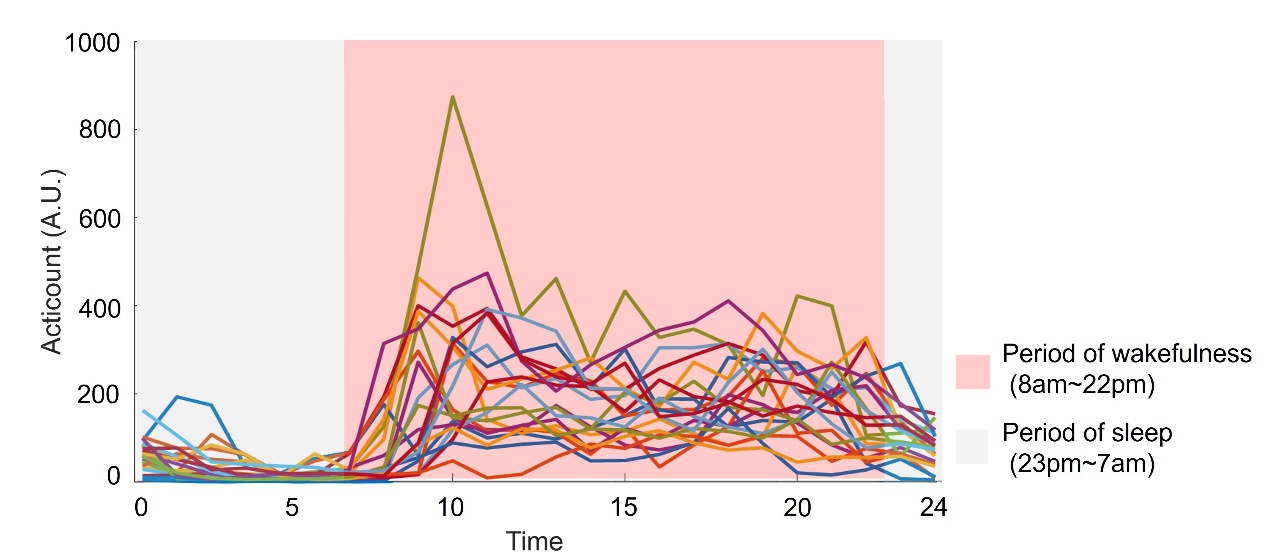

Supplement: Multimedia Appendix 3 [file jmir_v27i1e63897_app3.docx]
