# Supplementary material for: The Prognostic Significance of Sleep and Circadian Rhythm for Myocardial Infarction Outcomes: Case-Control Study
Source: J Med Internet Res. 2025 Feb 4;27:e63897. doi: 10.2196/63897 (PMC11836589; doi:10.2196/63897)
Supplement: Multimedia Appendix 4 [file jmir_v27i1e63897_app4.docx]

**Multimedia Appendix 4.** Correlations of the parameters of actigraphy and prognosis of patients with myocardial infarction after adjusting for age and gender.

| (n=28) | Total days of current admission | Any  re-admission | Any  ICU | Times of re-admission | Total days of re-admission | Any catheterization | Mean Days of re-admission |
| --- | --- | --- | --- | --- | --- | --- | --- |
| TST | -0.185 | 0.172 | 0.1 | 0.234 | 0.103 | 0.261 | -0.009 |
| SOL | -0.102 | 0.075 | -0.181 | 0.067 | -0.09 | 0.247 | -0.136 |
| SE | 0.132 | 0.33 | 0.17 | 0.362 | 0.347 | 0.18 | 0.352 |
| WASO | -0.09 | 0.242 | -0.079 | 0.189 | -0.108 | 0.242 | -0.122 |
| Awake | -0.328 | 0.063 | 0.061 | 0.065 | -0.197 | 0.152 | -0.304 |
| **Activity counts** |  |  |  |  |  |  |  |
| Daytime Act | 0.362 | -0.043 | -0.299 | -0.219 | -0.263 | -0.28 | -0.082 |
| Daytime Act _std_ | 0.208 | -0.147 | -0.335 | -0.294 | -0.316 | -0.324 | -0.187 |
| Night Act | 0.560^**^ | 0.035 | -0.075 | -0.071 | -0.036 | -0.136 | 0.128 |
| Night Act _std_ | 0.353 | -0.227 | -0.275 | -0.325 | -0.232 | -0.313 | -0.112 |
| **Active–Rest rhythm** |  |  |  |  |  |  |  |
| M10 | 0.385 | -0.123 | -0.317 | -0.288 | -0.288 | -0.336 | -0.114 |
| Time of M10 (pm) | -0.036 | 0.373 | 0.457^*^ | 0.527^**^ | 0.579^**^ | 0.202 | 0.466^*^ |
| L5 | 0.381 | -0.012 | -0.185 | -0.159 | -0.237 | -0.059 | -0.108 |
| Time of L5 (am) | 0.23 | 0.124 | 0.134 | 0.163 | 0.239 | -0.034 | 0.215 |
| RA | -0.074 | -0.108 | -0.067 | -0.071 | 0.031 | -0.195 | 0.04 |
| IV | -0.133 | 0.263 | 0.31 | 0.334 | 0.300 | 0.109 | 0.224 |
| IS | -0.363 | -0.154 | -0.31 | -0.268 | -0.423^*^ | -0.15 | -0.413^*^ |
| Dichotomy Index | -0.358 | -0.149 | 0.036 | 0.001 | 0.143 | -0.292 | 0.008 |
| Results are expressed as ^*^*P-*value < 0.05, ^**^ *P*-value < 0.01 using partial correlation coefficient (r) analysis, after adjusting gender and age.  Act: activity; Awake: number of awakenings; IS: interdaily stability; IV: intradaily variability; L5: averaged least active 5 consecutive hours; M10: averaged most active 10 consecutive hours; MI: myocardial infarction; RA: relative amplitude; SE: sleep efficiency; SOL: sleep onset latency; std: standard deviation; TST: total sleep time; WASO: wake after sleep onset. | | | | | | | |
