# Supplementary material for: The Prognostic Significance of Sleep and Circadian Rhythm for Myocardial Infarction Outcomes: Case-Control Study
Source: J Med Internet Res. 2025 Feb 4;27:e63897. doi: 10.2196/63897 (PMC11836589; doi:10.2196/63897)
Supplement: Multimedia Appendix 5 [file jmir_v27i1e63897_app5.docx]

**Multimedia Appendix 5.** Correlations of the parameters of heart rate variability and prognosis of patients with myocardial infarction after adjusting for age and gender.

| (n=22) | Total days of current admission | Any  re-admission | Any  ICU | Times of re-admission | Total days of re-admission | Any catheterization | Mean Days of re-admission |
| --- | --- | --- | --- | --- | --- | --- | --- |
| Mean | 0.043 | -0.234 | -0.058 | -0.234 | -0.144 | -0.108 | -0.144 |
| HR | -0.054 | 0.236 | 0.043 | 0.236 | 0.135 | 0.102 | 0.135 |
| SDRR | 0.197 | -0.390 | 0.010 | -0.390 | -0.363 | -0.274 | -0.363 |
| RMSSD | -0.025 | -0.304 | -0.110 | -0.304 | -0.247 | -0.090 | -0.247 |
| pNN50 | 0.073 | -0.200 | -0.074 | -0.200 | -0.164 | -0.073 | -0.164 |
| pNN20 | -0.016 | -0.321 | -0.145 | -0.321 | -0.274 | -0.039 | -0.274 |
| SD1 | -0.025 | -0.304 | -0.110 | -0.304 | -0.247 | -0.090 | -0.247 |
| SD2 | 0.206 | -0.392 | 0.018 | -0.392 | -0.368 | -0.284 | -0.368 |
| SD ratio | -0.106 | 0.341 | -0.293 | 0.341 | 0.455^*^ | 0.456^*^ | 0.455^*^ |
| DC value | -0.109 | -0.385 | -0.082 | -0.385 | -0.329 | -0.226 | -0.329 |
| Results are expressed as ^*^ *P-*value < 0.05, ^**^ *P*-value < 0.01 using partial correlation coefficient (r) analysis, after adjusting gender and age.  DC value: deceleration capacity of heart rate; HR: average heart rate; HRV: heart rate variability; Mean: mean RR intervals; MI: myocardial infarction; pNN20: percentage of successive RR intervals that differ by more than 20 ms; pNN50: percentage of successive RR intervals that differ by more than 50 ms; RMSSD: root mean square of successive differences; SD ratio: Ratio of SD1-to-SD2; SD1: Poincaré plot standard deviation perpendicular the line of identity; SD2: Poincaré plot standard deviation along the line of identity; SDRR: standard deviation of the RR interval. | | | | | | | |
